# Housing Instability and Type 2 Diabetes Outcomes

**DOI:** 10.1001/jamanetworkopen.2025.4852

**Published:** 2025-04-14

**Authors:** Seth A. Berkowitz, Aileen Ochoa, Marlena L. Kuhn, Jenine Dankovchik, Jenna M. Donovan, Myklynn LaPoint, Mufeng Gao, Sanjay Basu, Michael G. Hudgens, Rachel Gold

**Affiliations:** 1 Division of General Medicine and Clinical Epidemiology, Department of Medicine, University of North Carolina at Chapel Hill School of Medicine, Chapel Hill; 2 Center for Health Promotion and Disease Prevention, University of North Carolina at Chapel Hill School of Medicine, Chapel Hill; 3 Cecil G. Sheps Center for Health Services Research, University of North Carolina at Chapel Hill, Chapel Hill; 4 Department of Research, OCHIN, Portland, Oregon; 5 Department of Social Medicine, Center for Health Equity Research, University of North Carolina at Chapel Hill, Chapel Hill; 6 Department of Biostatistics, University of North Carolina at Chapel Hill, Chapel Hill; 7 Clinical Product Development, Waymark Care, San Francisco, California; 8 Kaiser Permanente Northwest Center for Health Research, Portland, Oregon

## Abstract

**Importance:**

Housing instability may worsen control of type 2 diabetes outcomes.

**Objective:**

To estimate the association between stable vs unstable housing and type 2 diabetes outcomes.

**Design, Setting, and Participants:**

This cohort study analyzed electronic health record data of adults with type 2 diabetes from US community-based health centers from June 2016 to April 2023. Targeted minimum loss estimation was used to examine the association between having vs not having housing instability and subsequent type 2 diabetes outcomes, adjusting for age, date of housing instability assessment, sex, race and ethnicity (social constructs that may indicate the experience of racism), language, comorbidities, health insurance, income, and census-tract level social vulnerability index. Analyses were conducted from July 2023 to September 2024.

**Exposure:**

Report of housing stability or instability.

**Main Outcomes and Measures:**

Mean hemoglobin A_1c_ (HbA_1c_) level was the primary outcome; secondary outcomes included systolic and diastolic blood pressure (SBP and DBP) and low-density lipoprotein (LDL) cholesterol. The primary time point was 12 months after initial assessment, with secondary time points at 6, 18, 24, 30, and 36 months.

**Results:**

A total of 90 233 individuals were included (mean [SD] age, 55.4 [13.7] years; 50 772 female [56.3%]; 25 602 Black [28.4%], 27 277 Hispanic [31.4%], 51 720 White [57.3%]); 28 784 individuals (31.9%) had a primary language other than English, and 15 713 (17.4%) reported housing instability. Prior to first housing instability assessment, mean (SD) HbA_1c_ was 7.64% (1.94%), mean (SD) SBP was 130.0 (13.5) mm Hg, mean (SD) DBP was 78.2 (7.8) mm Hg, and mean (SD) LDL cholesterol was 101.1 (35.2) mg/dL. We estimated had all individuals experienced stable housing, compared with unstable housing, mean HbA_1c_ would have been 0.12% lower (95% CI, −0.16% to −0.07%; *P* < .001), SBP would have been 0.77 mm Hg lower (95% CI, −1.14 mm Hg to −0.39 mm Hg; *P* < .001), and DBP 0.27 mm Hg lower (95% CI, −0.49 mm Hg to −0.06 mm Hg; *P* = .01), but LDL cholesterol would not have been lower (estimated difference, −1.46 mg/dL, 95% CI, −2.96 mg/dL to 0.03 mg/dL; *P* = .05) at 12 months, with numerically similar results at other time points.

**Conclusions and Relevance:**

In this cohort study, housing stability was associated with small differences in type 2 diabetes outcomes; combining housing stability interventions with other diabetes interventions may be needed to improve type 2 diabetes outcomes more substantially.

## Key Points

**Question:**

Is housing instability associated with worse type 2 diabetes outcomes?

**Findings:**

This cohort study including 90 233 adults with type 2 diabetes found that housing instability was associated with modestly worse hemoglobin A_1c_ and systolic and diastolic blood pressure values, but not with worse low-density lipoprotein cholesterol.

**Meaning:**

The association of housing instability with modestly worse diabetes outcomes suggests that interventions to address housing instability may need to be combined with interventions that address other barriers to optimal diabetes care to have large impacts on diabetes outcomes.

## Teaser

This cohort study of US adults with type 2 diabetes examines the association of housing instability with health outcomes, including hemoglobin A_1c_, blood pressure, and cholesterol levels.

## Introduction

Housing instability refers to several related hardships, including homelessness, facing eviction, frequent moves, overcrowding, difficulty paying rent, and taking up residence with friends or relatives.^1,2,3^ Housing instability has been associated with numerous poor health outcomes including greater emergency department utilization, poor mental health, and suboptimal child development.^1,4,5,6,7,8^ Given these associations, reducing housing instability is a priority goal of Healthy People 2030.^9^

In the context of type 2 diabetes, housing instability could worsen health through several pathways, including forced trade-offs between paying for housing and medications,^10^ disrupting continuity of care and interfering with self-care activities by causing stress and life disruptions,^11,12,13^ worsening mental health, and pushing people to live in areas with few resources needed for health.^14^ These consequences could lead to greater levels of cardiometabolic risk factors such as hemoglobin A_1c_ (HbA_1c_), blood pressure, and low-density lipoprotein (LDL) cholesterol, which are associated with long-term diabetes complications.^2,11,15,16,17,18,19,20,21^ However, despite the plausibility of these associations, there has been little prior quantification of their magnitude, and most previous work has been cross-sectional, meaning it is not clear how much of an improvement in type 2 diabetes outcomes might be possible from addressing housing instability.^14,22^ Thus, to help inform possible interventions, there is a need to better quantify, using longitudinal data, the extent to which addressing housing instability might improve type 2 diabetes outcomes.

This study examines the association between housing instability and diabetes outcomes in a large observational cohort of individuals seen in a community-based health center network. We tested the hypothesis that stable housing would be associated with better outcomes than unstable housing using a target trial emulation framework.^23,24,25^ This approach structures the analyses as a comparison between having unstable housing and having stable housing, as if these situations could be assigned by researchers. Of course, we would never assign people to experience unstable housing, and we are not studying an actual intervention. Instead, we use the conceptual approach of designing this observational study to emulate a clinical trial that would answer the question of interest to help avoid biases common in observational studies, like misaligning the start of exposure and follow-up.^23^ Study results can help guide future interventional work by shedding light on what impact actual housing interventions may have on diabetes outcomes. They may also illuminate what sample sizes may be needed to detect such impacts, and over what time frame such impacts could become apparent.

## Methods

This observational longitudinal cohort study used a target trial emulation approach^26^ to quantify the connection between housing instability and type 2 diabetes outcomes. The study period was June 2016 to April 2023 and analyses were conducted from July 2023 to September 2024. The University of North Carolina at Chapel Hill institutional review board approved this study with an exemption for providing written informed consent as it was a retrospective review of data collected for routine clinical operations, without contacting individuals. This manuscript follows the Strengthening the Reporting of Observational Studies in Epidemiology (STROBE) reporting guideline for cohort studies.

### Target Trial Protocol

The target trial emulation method involves specifying a protocol for a hypothetical trial (eTable 1 in Supplement 1).^24,27^ Study data came from community-based health centers, located across the US, that use the same electronic health record (EHR) through membership in OCHIN, Inc, a national nonprofit health information technology company.^28,29^ With these data, we used previously validated methods to construct a cohort of adults (age 18 years and older) who underwent housing instability assessment and had type 2 diabetes.^29,30,31^ Tools used to assess housing instability within the EHR data included the Accountable Health Communities Health-Related Social Needs Screening tool and the Protocol for Responding to and Assessing Patient’s Assets, Risks, and Experience (PRAPARE).^29,32,33,34,35^ In prior work, several housing instability assessment tools have been shown to assess housing instability similarly well.^29,35^A positive response to any housing instability item was taken to indicate housing instability.

There were 2 scenarios we sought to emulate. The primary scenario was one in which a hypothetical intervention could prevent housing instability—that is, make it so people would never report housing instability during the study period. To study this scenario, individuals were assigned to a “no housing instability” exposure group if they reported not experiencing housing instability at their initial housing stability status assessment, and were assigned to the “housing instability” exposure group if they reported housing instability at their initial housing stability assessment. Housing stability is a time-varying exposure, and so while individuals could be categorized as belonging to a particular exposure group at their initial housing stability assessment, they may not have adhered to that exposure over time.

The secondary scenario we sought to emulate was a hypothetical intervention treating housing instability once it occurred. To investigate this secondary scenario, we created a subcohort of the main cohort by selecting those individuals who reported housing instability and had a reassessment within 12 months of reporting housing instability. Those who reported no longer experiencing housing instability were categorized into the “resolved housing instability” group, while those who again reported housing instability were categorized into the “continue to experience housing instability” group.

This study’s primary outcome was HbA_1c_. We also examined secondary outcomes of systolic and diastolic blood pressure (SBP and DBP) and LDL cholesterol. Time zero in these analyses (equivalent to the date an individual became eligible for, enrolled in, and was assigned a treatment strategy in an actual clinical trial^23^) was set at the time of first recorded housing instability assessment (for the prevention strategy) and the time of first housing instability assessment within 12 months after a positive housing instability assessment (for the treatment strategy) (eFigure 1 in Supplement 1). The primary outcome time point was 12 months after time zero. For the prevention strategy, we also examined secondary time points of 6, 18, 24, 30, and 36 months after time zero.

We sought to estimate a difference in mean outcomes had participants experienced (vs not experienced) housing instability according to the 2 scenarios described above. If the difference in means estimated had a causal interpretation, it would correspond to an average treatment effect (ATE) estimand. However, we caution that whether the assumptions needed for causal inference have been met in this observational study cannot be known.

Because this is an observational study, we considered factors that could confound the association between housing instability and diabetes outcomes, selected based on past research.^2,8,11,14,20^ These were: age at first housing instability assessment, date of first housing instability assessment, sex, preferred language, health insurance, income (as percentage of the federal poverty threshold in the year measured to account for inflation and household size), census-tract level social vulnerability index (SVI),^36^ and prior values of the outcome (eg, if the outcome was HbA_1c_ at 12 months, prior HbA_1c_ values were considered covariates). Another covariate was race and ethnicity, which was included as these socially constructed categories may serve as a proxy for discrimination at multiple levels and in multiple settings along the conceptual framework of housing instability and clinical outcomes. Race and ethnicity data came from electronic health record data, which did not indicate whether these data were self-reported. Categories included American Indian or Alaska Native, Asian, Black, Native Hawaiian or other Pacific Islander, White, multiple, and not reported. Finally, we adjusted for an *International Statistical Classification of Diseases and Related Health Problems, Tenth Revision (ICD-10)* code based comorbidity index that combines the Charlson and Elixhauser indices and has been associated with mortality and health care utilization in community-dwelling adults.^37^ The comorbidity index was constructed using *ICD-10* diagnostic codes over the year prior to first housing instability assessment. The health insurance, income, SVI, and prior outcome values were treated as time-varying, and the other variables were time-fixed. A directed acyclic graph (DAG) depicts the assumptions we made about variable interrelationships (eFigure 2 in Supplement 1).

Individuals in the study could be censored for 2 reasons: crossover and loss to follow-up. Crossover was defined as not adhering to the initially assigned treatment strategy (eg, reporting housing instability after being assigned to the no housing instability group). Once censored, data were used to estimate the censoring mechanism, but not for outcome analyses after the point of censoring. This approach is appropriate because individuals are no longer informative regarding the association between their assigned treatment strategy and study outcomes once they stop following the strategy.^23,38,39^ If data from such individuals are used for outcome analysis, the data could bias estimates of the treatment strategy of interest. However, if those data are removed entirely, that could also create bias, as the data represent a nonrandom subset of data from the assigned group. Therefore, censoring data at the point of crossover and estimating the censoring mechanism can reduce bias.^23,38,39^

Loss-to-follow-up was defined as not having a housing instability assessment at least every 24 months, or not having an outcome assessment at some point in the preceding 6 months for a given time point. Individuals censored for one analysis could be uncensored for other analyses. For example, an individual who did not have an HbA_1c_ for in the 6 months before the 12-month time point but did have a SBP assessment in the preceding 6 months would be considered censored for the 12-month HbA_1c_ analysis but uncensored for the 12-month SBP analysis. And an individual who had a crossover event 14 months after the start of follow-up could be uncensored for the 12-month analyses but censored for the 18-month (and later) analyses. Our analytic approach accounted for censoring through estimation of the censoring mechanism, so data from individuals who were censored was still used in the analysis to estimate the censoring mechanism. Missing data other than that which resulted in censoring was addressed through multiple imputation by chained equations, with 50 imputed datasets.^40,41^

### Statistical Analysis

We used targeted minimum loss estimation (TMLE) to contrast the treatment strategies of interest.^42,43,44^ TMLE is a multiply robust approach that, at each time point, begins by estimating an outcome model, and uses this model to estimate study outcomes under the scenarios of interest.^44,45^ The next step is to estimate the propensity score (ie, the probability of being in the treated group, based on the included covariates). The estimated propensity score is then transformed into a covariate in a new model that updates the initial outcome estimate. Including the covariate derived from the propensity score helps remove residual bias that was not accounted for in the initial outcome model. Moreover, in its estimation of the propensity score, TMLE also accounts for possibly informative censoring (eg, when the reason for missing data is related to the outcome of interest). This works analogously to inverse probability of censoring weighting, whereby observed characteristics are used to estimate the probability of censoring.

The primary TMLE approach used generalized linear regression models, but TMLE can also incorporate machine learning methods ensembled together as what van der Laan et al^46^ have called a “SuperLearner.” This can reduce the risk of model misspecification and account for nonlinearities in the data generating process, but is computationally intensive, especially at longer time points. In sensitivity analyses, we conducted TMLE using an ensemble of generalized linear models, the XGBoost approach to gradient boosting, and multivariable adaptive regression splines.

As a sensitivity analysis to investigate whether housing instability may have been associated with particularly worse outcomes for individuals using insulin, we repeated the primary analyses but in a cohort restricted to individuals documented in the EHR to have been prescribed insulin prior to their initial housing instability assessment. To investigate whether results may been sensitive to the choice of follow-up window, we repeated the main analyses, but considered individuals lost to follow-up and censored if they did not report housing stability status at least every 12 months (as opposed to every 24 months in the main analyses). Finally, as a sensitivity analysis to assess the potential impact of other health-related social needs, we repeated the main analyses but additionally adjusted for food and transportation needs as reported along with housing stability status.

For analysis, we used SAS version 9.4 (SAS Institute) and R version 4.3.1 (R Project for Statistical Computing). Key R packages were mice for multiple imputation, and lmtp for the TMLE analyses.^47,48^ After estimating results in each of the multiply imputed datasets, we combined estimates using Rubin rules.^49^ The threshold for statistical significance was *P* < .05, using 2-sided tests.

## Results

Of 442 154 adults with type 2 diabetes, 90 233 were assessed for housing instability at least once between June 2016 to April 2023 (eTable 2 in Supplement 1). Of the 90 233 adults with type 2 diabetes included in this study, the mean (SD) age was 55.4 (13.7) years (Table 1). By demographic characteristics, 50 772 individuals (56.3%) were female; 25 602 (28.4%) identified as Black, 27 277 (31.4%) as Hispanic, and 51 720 (57.3%) as White; 28 784 individuals (31.9%) had a primary language other than English. Participants resided in 41 states and were seen in 1231 unique clinical departments. There were 15 713 (17.4%) who reported housing instability on initial assessment, while 74 520 (82.6%) reported stable housing.

**Table 1.** Demographic Characteristics of Cohort

Characteristic	Individuals, No. (%)			*P* value
	Overall (n = 90 233)	No housing instability (n = 74 520)	Housing instability (n = 15 713)	
Age at initial housing instability assessment, mean (SD), y	55.4 (13.7)	56.0 (13.9)	52.7 (11.8)	<.001
Sex				
Female	50 772 (56.3)	42 582 (57.2)	8190 (52.2)	<.001
Male	39 440 (43.7)	31 925 (42.8)	7515 (47.8)	
Racial identity				
American Indian or Alaska Native	1004 (1.1)	785 (1.1)	219 (1.4)	<.001
Asian	3839 (4.3)	3527 (4.7)	312 (2.0)	
Black	25 602 (28.4)	21 239 (28.5)	4363 (27.8)	
Multiple	759 (0.8)	550 (0.7)	209 (1.3)	
Native Hawaiian or other Pacific Islander	691 (0.8)	573 (0.8)	118 (0.8)	
White	51 720 (57.3)	42 533 (57.1)	58.47% (9187)	
Not reported^a^	6618 (7.3)	5313 (7.1)	1305 (8.3)	
Hispanic ethnicity	27 277 (31.4)	22 540 (31.4)	4737 (31.3)	.80
Primary language other than English^b^	28 784 (31.9)	24 579 (33.0)	4205 (26.8)	<.001
Comorbidity index, mean (SD)	0.22 (0.94)	0.21 (0.95)	0.27 (0.88)	<.001
Health insurance				
Medicaid	29 859 (33.7)	23 032 (31.5)	6827 (44.4)	<.001
Medicare	23 520 (26.6)	20 789 (28.4)	2731 (17.8)	
Other public	1916 (2.2)	1555 (2.1)	361 (2.4)	
Private	16 369 (18.5)	14 851 (20.3)	1518 (9.9)	
Uninsured	16 892 (19.1)	12 967 (17.7)	3925 (25.6)	
Household income as percentage of federal poverty threshold, mean (SD), %	113.7 (256.5)	120.8 (270.3)	83.0 (182.1)	<.001
Social vulnerability index at census tract level, mean (SD)^c^	0.69 (0.24)	0.69 (0.25)	0.71 (0.23)	<.001
HbA_1c_ in year prior to housing instability assessment, mean (SD), %	7.64 (1.94)	7.58 (1.88)	7.91 (2.17)	<.001
SBP in year prior to housing instability assessment, mean (SD), mm Hg	130.01 (13.52)	129.97 (13.38)	130.23 (14.20)	.69
DBP in year prior to housing instability assessment, mean (SD), mm Hg	78.23 (7.97)	78.00 (7.87)	79.36 (8.32)	<.001
LDL cholesterol in year prior to housing instability assessment, mean (SD), mg/dL	101.07 (35.24)	100.84 (35.14)	102.23 (35.72)	<.001

Abbreviations: DBP, diastolic blood pressure; HbA_1c_, hemoglobin A_1c_; LDL, low density lipoprotein; SBP, systolic blood pressure.

SI conversion factor: To convert HbA_1c_ to proportion of total hemoglobin, multiply by 0.01; LDL cholesterol to millimoles per liter, multiply by 0.0259.

^a^ Not reported for the racial identity variables indicates that a racial identity was not reported in the electronic health record data used for this study.

^b^ The 5 most common languages other than English were Spanish, Chinese (variety not specified), Vietnamese, French, and Russian.

^c^ Greater social vulnerability index scores indicate greater risk.

In the year prior to first housing instability assessment, mean (SD) HbA_1c_ was 7.64% (1.94) (to convert to proportion of total hemoglobin, multiply by 0.01), mean (SD) SBP was 130.0 (13.5) mm Hg, mean (SD) DBP was 78.2 (7.8) mm Hg, and mean (SD) LDL cholesterol was 101.1 (35.2) mg/dL (to convert to millimoles per liter, multiply by 0.0259). In analyses that estimated the impact of preventing housing instability, we estimated that had all individuals experienced stable housing, mean HbA_1c_ would have been 7.58% at 12 months (95% CI, 7.56% to 7.60%), and had all individuals experienced housing instability, mean HbA_1c_ would have been 7.70% at 12 months (95% CI, 7.66% to 7.75%) (Figure; eTable 3 in Supplement 1). This corresponded to an estimated difference in means at 12 months of 0.12% lower HbA_1c_ (95% CI, −0.16% to −0.07%; *P* < .001), had everyone had stable, compared with unstable, housing (Table 2). Results at other time points were similar.

**Figure.**
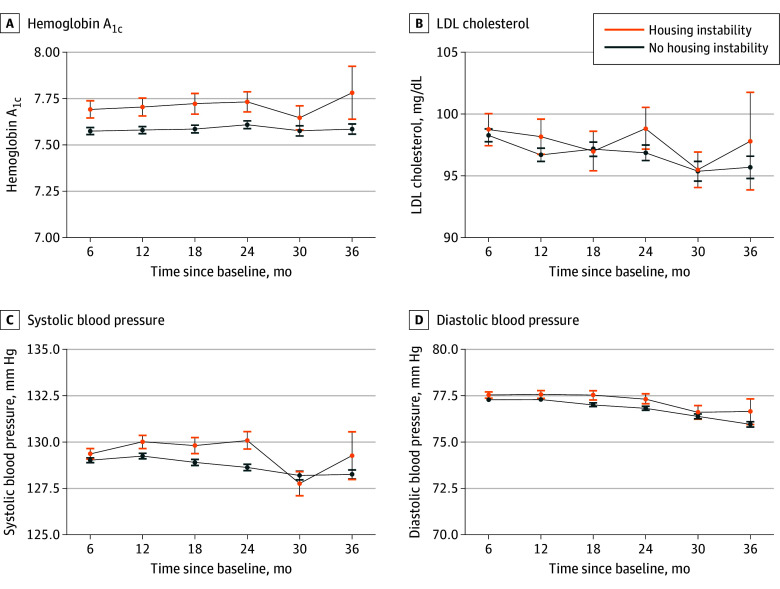
Estimated Mean Hemoglobin A_1c_, Blood Pressure, and Low-Density Lipoprotein (LDL) Cholesterol for Individuals Experiencing Housing Instability SI conversion factor: To convert hemoglobin A_1c_ to proportion of total hemoglobin, multiply by 0.01; LDL cholesterol to millimoles per liter, multiply by 0.0259.

**Table 2.** Estimated Differences in Means Under the Prevent Housing Instability Strategy

Time point	Hemoglobin A_1c_		Systolic blood pressure		Diastolic blood pressure		LDL cholesterol	
	Estimated difference in mean, % (95% CI)^a^	*P* value	Estimated difference in mean, mm Hg (95% CI)^a^	*P* value	Estimated difference in mean, mm Hg (95% CI)^a^	*P* value	Estimated difference in mean, mg/dL (95% CI)^a^	*P* value
6 mo	−0.12 (−0.16 to −0.07)	<.001	−0.34 (−0.64 to −0.04)	.03	−0.24 (−0.41 to −0.06)	.009	−0.46 (−1.84 to 0.91)	.51
12 mo	−0.12 (−0.17 to −0.07)	<.001	−0.77 (−1.14 to −0.39)	.001	−0.27 (−0.49 to −0.06)	.01	−1.46 (−2.96 to 0.03)	.05
18 mo	−0.14 (−0.19 to −0.08)	<.001	−0.91 (−1.36 to −0.45)	.001	−0.51 (−0.77 to −0.25)	.001	0.14 (−1.54 to 1.82)	.87
24 mo	−0.12 (−0.18 to −0.07)	<.001	−1.45 (−1.93 to −0.97)	<.001	−0.49 (−0.77 to −0.21)	<.001	−1.97 (−3.76 to −0.18)	.03
30 mo	−0.07 (−0.14 to 0.00)	.04	0.43 (−0.25 to 1.11)	.21	−0.22 (−0.61 to 0.17)	.26	−0.12 (−1.73 to 1.49)	.88
36 mo	−0.20 (−0.34 to −0.05)	.007	−1.02 (−2.31 to 0.26)	.12	−0.68 (−1.37 to 0.02)	.06	−2.11 (−6.17 to 1.95)	.31

Abbreviation: LDL, low-density lipoprotein.

SI conversion factor: To convert hemoglobin A_1c_ to proportion of total hemoglobin, multiply by 0.01; LDL cholesterol to millimoles per liter, multiply by 0.0259.

^a^ Estimated difference in mean compares the estimated difference in the outcomes between counterfactual scenarios in which individuals did not vs did experience housing instability from the time of first assessment to the specified time point. A negative value indicates estimated benefit for preventing housing instability. The differences in means were estimated using a longitudinal targeted minimum loss estimation approach.

For blood pressure outcomes, the difference in means at 12 months were −0.8 mm Hg (95% CI, −1.1 mm Hg to −0.4 mm Hg; *P* < .001) for SBP and −0.3 mm Hg (95% CI, −0.5 mm Hg to −0.1 mm Hg; *P* = .01) for DBP. At other time points, estimated differences in means were small, but the 95% confidence interval excluded zero for SBP and DBP at 6, 18, and 24 months, with less precise estimates at 30 and 36 months. For LDL cholesterol, the difference in means at 12 months was −1.5 mg/dL (95% CI, −3.0 mg/dL to 0.0 mg/dL; *P* = .05) and differences in means at other time points were similarly small in magnitude, with 95% confidence intervals mostly including zero.

Results of sensitivity analyses using a SuperLearner approach for estimation were similar to those of the main analytic approach (eTable 4 in Supplement 1). To have 80% or greater power to detect a difference in HbA_1c_ of the magnitude observed in this study in an actual randomized clinical trial, assuming a similar standard deviation, a sample size of 3808 participants per arm, or 7616 total participants, would be required.

There were 3718 individuals in the cohort used to evaluate the strategy of treating housing instability once it developed (eTables 5 and 6 in Supplement 1). Analyses in this cohort did not estimate a significant difference in means for HbA_1c_ (−0.04%; 95% CI, −0.24% to 0.16%) or other study outcomes at 12 months, although results in this cohort were less precise than in the main cohort given the smaller sample size (Table 3). Results of sensitivity analyses restricted to individuals prescribed insulin, using a 12-month, rather than a 24-month, loss to follow-up window, or additionally adjusting food and transportation needs, were not meaningfully different from the main results (eTables 7 through 9 in Supplement 1).

**Table 3.** Estimated Difference in Means Under the Treat Housing Instability After It Occurs Strategy at 12 Months

Variable	Hemoglobin A_1c_, % (95% CI)	Mean estimate, (95% CI)		
		Systolic blood pressure, mm Hg	Diastolic blood pressure, mm Hg	LDL cholesterol, mg/dL
No housing instability	7.72 (7.59 to 7.84)	128.34 (127.33 to 129.35)	77.28 (76.70 to 77.87)	93.90 (90.59 to 97.22)
Experiencing housing instability	7.76 (7.59 to 7.93)	129.56 (128.42 to 130.70)	78.37 (77.70 to 79.04)	96.99 (91.50 to 102.47)
Estimated difference in means^a^	−0.04 (−0.24 to 0.16)	−1.22 (−2.70 to 0.26)	−1.09 (−1.94 to −0.24)	−3.09 (−9.42 to 3.25)

Abbreviation: LDL, low-density lipoprotein.

SI conversion factor: To convert hemoglobin A_1c_ to proportion of total hemoglobin, multiply by 0.01; LDL cholesterol to millimoles per liter, multiply by 0.0259.

^a^ Estimated difference in mean compares the estimated difference in the outcomes between counterfactual scenarios in which individuals did not vs did experience housing instability from the time of first assessment to the 12-month time point. A negative value indicates estimated benefit for preventing housing instability. The differences in means were estimated using a longitudinal targeted minimum loss estimation approach.

## Discussion

In this observational longitudinal cohort study of individuals seen in community-based health centers, we estimated that preventing housing instability, compared with experiencing housing instability, was associated with modestly better HbA_1c_ and blood pressure, but not LDL cholesterol. However, the magnitude of these differences may not be clinically meaningful. Study findings that estimated benefits of the hypothetical intervention were roughly similar at time points ranging from 6 to 24 months suggest that actual interventions may be able to detect benefits, if any, over a 6-to-12-month follow-up period, which is useful for intervention and trial planning.

These findings are consistent with and expand on those of prior work examining the association between housing instability and poor health in people with type 2 diabetes.^2,11,15,16,17,18,19,20,21^ Prior studies have shown housing instability to be associated with more emergency department visits and hospitalizations.^2^ Furthermore, some cross-sectional assessments have suggested associations with other poor diabetes outcomes.^14,19,20^ This study adds to previous work by quantifying more precisely what addressing housing instability may offer in terms of biomarkers of diabetes management.

The implications of these findings are noteworthy. As clinics and health plans seek to address health-related social needs, these findings may shape expectations concerning short-term biomarker changes that addressing housing instability may produce. One interpretation of these results is that stable housing may be an enabling condition for better diabetes outcomes—that is, it facilitates the self-care and clinical management activities diabetes requires—but is not itself sufficient for optimal diabetes care. Policy interventions could layer additional supports on top of the foundation that stable housing provides. For example, permanent supportive housing interventions can reduce housing instability, and from that base provide additional components that improve access to health care, support self-management activities, and forge long-term relationships with care providers.^50^ Such approaches may have a greater impact on diabetes outcomes than addressing a single factor, like housing instability, alone. Indeed, a 2024 meta-analysis^51^ of multi-level type 2 diabetes interventions that addressed multiple pathways simultaneously did estimate benefits larger than those observed here. Therefore, the results presented here are best interpreted as a way to understand the potential impact on type 2 diabetes outcomes of addressing one specific pathway—housing instability—that might be difficult to isolate in practice. It is also important to consider that improving type 2 diabetes outcomes is not the primary reason to address housing instability. Ensuring stable housing is a worthwhile goal in its own right, regardless of any health benefits that might result.

A clearly needed direction for future research is to test, using randomized trials, the health effects of interventions that include improving housing stability. The findings of this study support conducting such trials, illustrate how much impact such interventions may be expected to have on their own, and suggest that interventions to improve diabetes outcomes by addressing housing stability might be paired with interventions affecting other pathways to improved health to have greater impact. Another important direction for future research is to better understand the mechanisms through which housing stability affects type 2 diabetes outcomes. Although these are broadly understood, there are likely important nuances in particular communities. For example, for individuals who prefer a language other than English, impacts of housing instability such as continuity of care disruptions and forced trade-offs between affording housing and medications may interrelate with other important barriers to type 2 diabetes care, such as language barriers and the impact of documentation status.^52,53^ Future research should investigate these important interrelationships.

### Strengths and Limitations

This study had several strengths. First, we utilized a large cohort followed longitudinally with representation from throughout the US. Next, this dataset had a large proportion of individuals who prefer a language other than English. Finally, the approach used allowed us to better understand the association between housing instability and poor health in a way that could not be tested otherwise.

Study findings should be interpreted with reference to several limitations. Most importantly, this was a nonrandomized, observational study using clinical records data, and is thus subject to potential confounding and selection bias. However, our approach did take several steps to mitigate such risks, accounting for a large set of measured confounders, using data both before and after the initial housing instability assessment to help account for unmeasured time-fixed confounders that affect experiencing housing instability, and using both study design (eg, target trial emulation) and analytic approaches (eg, multiple imputation and estimation of the censoring mechanism) that help account for selection bias. Furthermore, we did not have data on some factors that may impact financial stability, such as marital status. Next, this was an emulation of a hypothetical intervention, not a test of an actual intervention. In this emulation, we compared scenarios in which individuals experienced housing instability continuously to scenarios in which they did not experience housing instability. However, individuals can also experience housing instability transiently.^54^ We would expect that this may have a different association with diabetes outcomes, likely associated with worse outcomes than not experiencing housing instability at all, but perhaps better outcomes than experiencing housing instability continuously, but we did not investigate that in this study.

## Conclusions

The health harms that unstable housing brings are one of many important reasons to ensure stable housing for all individuals. Study results help quantify the associations between unstable housing and one aspect of poor health—type 2 diabetes outcomes—and could inform subsequent studies seeking to address the threat to population health that housing instability represents.

## Notes

Supplement 1. **eFigure 1.** Covariate Assessment, Time 0, and Study Follow-Up **eFigure 2.** Directed Acyclic Graph **eTable 1.** Protocol for Target Trial and Comparison with Emulation Conducted **eTable 2.** Demographic Characteristics of Adults with Type 2 Diabetes Seen in OCHIN Network Who Did vs Did Not Undergo Housing Stability Assessment **eTable 3.** Estimated Mean Outcomes at Study Time Points Under Different Counterfactual Scenarios **eTable 4.** Comparison of Results at 12 Months Estimated Using SuperLearner and Generalized Linear Models **eTable 5.** Demographic Characteristics of Cohort that Reported Housing Instability and Were Reassessed within 12 Months **eTable 6.** Characteristics of Cohort Reporting Housing Instability and Reassessed within 12 Months Compared with Those Not Reassessed within 12 Months **eTable 7.** Estimated Differences in Means under the Prevention Strategy for Individuals Prescribed Insulin **eTable 8.** Estimated Differences in Means Under the Prevention Strategy with Loss to Follow-Up Window Shortened to 12 Months **eTable 9.** Estimated Differences in Means Under the Prevention Strategy with Adjustment for Food and Transportation Needs

Supplement 2.
Data Sharing Statement
