# Supplementary material for: Housing Instability and Type 2 Diabetes Outcomes
Source: JAMA Netw Open. 2025 Apr 14;8(4):e254852. doi: 10.1001/jamanetworkopen.2025.4852 (PMC11997728; doi:10.1001/jamanetworkopen.2025.4852)
Supplement: Supplement 1. — eFigure 1. Covariate Assessment, Time 0, and Study Follow-Up eFigure 2. Directed Acyclic Graph eTable 1. Protocol for Target Trial and Comparison with Emulation Conducted eTable 2. Demographic Characteristics of Adults with Type 2 Diabetes Seen in OCHIN Network Who Did vs Did Not Undergo Housing Stability Assessment eTable 3. Estimated Mean Outcomes at Study Time Points Under Different Counterfactual Scenarios eTable 4. Comparison of Results at 12 Months Estimated Using SuperLearner and Generalized Linear Models eTable 5. Demographic Characteristics of Cohort that Reported Housing Instability and Were Reassessed within 12 Months eTable 6. Characteristics of Cohort Reporting Housing Instability and Reassessed within 12 Months Compared with Those Not Reassessed within 12 Months eTable 7. Estimated Differences in Means under the Prevention Strategy for Individuals Prescribed Insulin eTable 8. Estimated Differences in Means Under the Prevention Strategy with Loss to Follow-Up Window Shortened to 12 Months eTable 9. Estimated Differences in Means Under the Prevention Strategy with Adjustment for Food and Transportation Needs [file jamanetwopen-e254852-s001.pdf]

Supplemental Online Content

Berkowitz SA, Ochoa A, Kuhn ML, et al. Housing instability and type 2 diabetes outcomes. *JAMA Netw Open.* 2025;8(4):e254852.
doi:10.1001/jamanetworkopen.2025.4852

eFigure 1. Covariate Assessment, Time 0, and Study Follow-Up

eFigure 2. Directed Acyclic Graph

eTable 1. Protocol for Target Trial and Comparison with Emulation Conducted

eTable 2. Demographic Characteristics of Adults with Type 2 Diabetes Seen in OCHIN Network Who Did vs Did Not Undergo Housing Stability Assessment

eTable 3. Estimated Mean Outcomes at Study Time Points Under Different Counterfactual Scenarios

eTable 4. Comparison of Results at 12 Months Estimated Using SuperLearner and Generalized Linear Models

eTable 5. Demographic Characteristics of Cohort that Reported Housing Instability and Were Reassessed within 12 Months

eTable 6. Characteristics of Cohort Reporting Housing Instability and Reassessed within 12 Months Compared with Those Not Reassessed within 12 Months

eTable 7. Estimated Differences in Means under the Prevention Strategy for Individuals Prescribed Insulin

eTable 8. Estimated Differences in Means Under the Prevention Strategy with Loss to Follow-Up Window Shortened to 12 Months

eTable 9. Estimated Differences in Means Under the Prevention Strategy with Adjustment for Food and Transportation Needs

This supplemental material has been provided by the authors to give readers additional information about their work.

© 2025 Berkowitz SA et al. *JAMA Network Open*.

eFigure 1. Covariate Assessment, Time 0, and Study Follow-Up

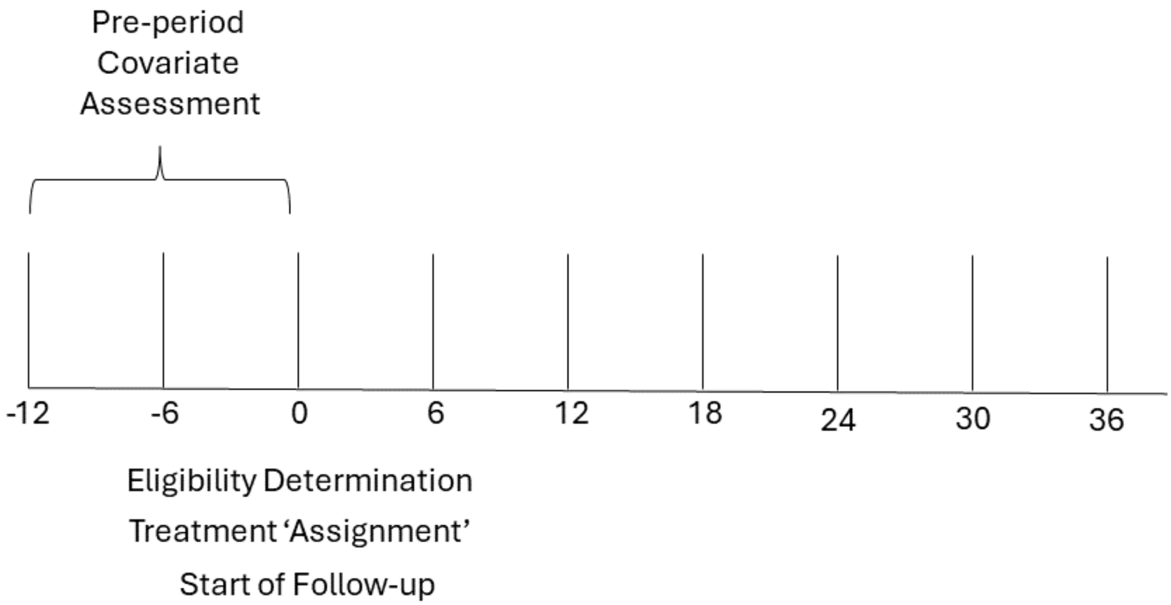

Appendix Figure 1 Legend: This figure depicts 'time 0' (the time when 'eligibility' began, treatment strategies were 'assigned', and the start of follow-up). Covariate assessment occurred during the 'pre-period', along with during study follow-up. Months are the time unit used.

© 2025 Berkowitz SA et al. *JAMA Network Open*.

eFigure 2. Directed Acyclic Graph

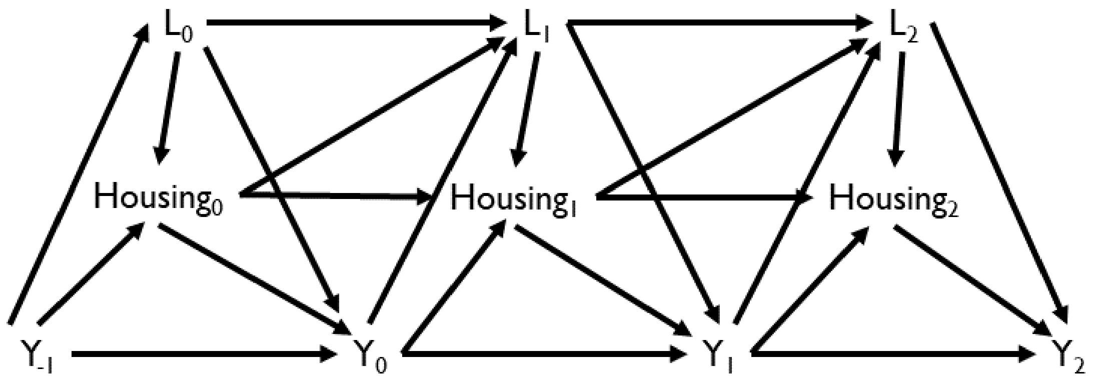

Appendix Figure 2 Legend: We conducted analyses under assumptions encoded in this Directed Acyclic Graph (DAG). Housing indicates an individual's housing stability (stable or unstable) at a given time point. Y indicates the study outcomes: hemoglobin a1c, systolic blood pressure, diastolic blood pressure, and LDL cholesterol. L indicates study covariates: age at first housing instability assessment, sex, race and ethnicity (socially constructed factors that may indicate the experience of racism), primary language, a comorbidity index, health insurance, income expressed as a percentage of the federal poverty threshold (to account for both household size and inflation), and the social vulnerability index of the census tract of residence. Numeric subscripts indicate the assessment time point, relative to the initial housing stability assessment (time 0). This DAG is useful for assessing conditional exchangeability, one of the key assumptions of causal inference analysis. Other assumptions include positivity and causal consistency. In this observational study, we cannot be sure that these assumptions hold, and thus we do not advocate a causal interpretation of the study results.

© 2025 Berkowitz SA et al. *JAMA Network Open*.

eTable 1. Protocol for Target Trial and Comparison with Emulation Conducted

Aspect of Protocol	Ideal Target Trial	Target Trial Emulation
Eligibility criteria	Age ≥ 18 years on date of first housing stability assessment	Same
	Diagnosis of type 2 diabetes	Same
	Housing stability assessment at participating clinic	Same
Treatment strategies	Experience housing instability	Same
	Do not experience housing instability	Same
Treatment assignment	Randomly assign treatment strategy at baseline	'Assign' treatment strategies based on their initial housing instability assessment response. Account for time fixed and time-varying covariates to help provide conditional exchangeability.
	Individuals and clinical team are aware of treatment assignment	Same
Outcomes	Hemoglobin A1c, Systolic Blood Pressure, Diastolic Blood Pressure, LDL Cholesterol	Same
Time Points	6 months, 12 months, 18 months, 24 months, 30 months, 36 months after initial housing instability assessment	Same
Causal Contrast/Estimand[1]	Effect of adhering to assigned rreatment ('per-protocol average treatment effect')	Same
Adherence/'cross-over'	Censor data at time of deviation from assigned treatment	Same
Loss to follow-up	For each eligible individual, follow-up starts at initial housing instability assessment and ends after 36 months, or administrative end of follow-up (April 30, 2023).	Same
	Individual considered lost to follow-up, and censored, for reason of adherence monitoring, 24 months after last housing instability assessment	Same
	Individual considered lost to follow-up, and censored, for reason of missing outcome assessment if there is no outcome assessment in the 6 months prior to the analytic time point (e.g., for 12-month analyses of HbA1c, no HbA1c assessment in months 7-12). Participants censored because of missing outcome data for an analysis of a given time point may be uncensored for analyses of a different time point (e.g., a	Same

© 2025 Berkowitz SA et al. *JAMA Network Open*.

	participant with no HbA1c assessment in months 7-12 but with HbA1c assessment in month 18 may be censored for analyses of the 12-month time point but uncensored for analyses for the 18-month time point)	
Missing covariate data	Try to minimize missing data through collection procedures, if missing data despite these efforts, use multiple imputation	Use multiple imputation
Statistical analysis	In an 'ideal' target trial with perfect adherence, no method of accounting for pre- and post-treatment factors associated with adherence and loss to follow-up would be needed	Censor individuals if and when they deviate from their assigned treatment strategy or are lost to follow-up, and use targeted minimum loss estimation to account for baseline covariates needed for conditional exchangeability, and to account for baseline and follow-up covariates associated with treatment strategy adherence and loss to follow-up to help prevent selection bias

[1] In an 'ideal' target trial, the intention-to-treat estimand would be equivalent to the per-protocol estimand as all participants would adhere to their assigned treatment.

Note: this trial protocol is adapted from Berkowitz SA, Ochoa A, Donovan JM, et al. Estimating the impact of addressing food needs on diabetes outcomes. *SSM - Popul Health*. 2024;27:101709. doi:10.1016/j.ssmph.2024.101709

© 2025 Berkowitz SA et al. *JAMA Network Open*.

eTable 2. Demographic Characteristics of Adults with Type 2 Diabetes Seen in OCHIN Network Who Did Vs. Did Not Undergo Housing Stability Assessment

Characteristic	Underwent Housing Stability Assessment	Did Not Undergo Housing Stability Assessment
	N = 90,233	N = 351,921
	Mean (SD) or % (N)	Mean (SD) or % (N)
Age, years	55.44 (13.65)	57.96 (14.36)
Female	56.28% (50,772)	54.59% (192,100)
Racial Identity		
American Indian/Alaska Native	1.11% (1,004)	1.12% (3,952)
Asian	4.25% (3,839)	7.36% (25,915)
Black	28.37% (25,602)	16.26% (57,222)
Multiple	0.84% (759)	0.68% (2,399)
Native Hawaiian or Other Pacific Islander	0.77% (691)	0.91% (3,208)
Not Reported	7.33% (6,618)	14.73% (51,846)
White	57.32% (51,720)	58.93% (207,379)
Hispanic Ethnicity	31.42% (27,277)	42.23% (148,606)
Primary Language Other Than English	31.92% (28,784)	
Health Insurance		
Medicaid	33.72% (29,859)	37.88% (133,252)
Medicare	26.56% (23,520)	25.34% (89,125)
Other Public	2.16% (1,916)	1.15% (40,48)
Private	18.48% (16,369)	14.79% (52,009)
Uninsured	19.07% (16,892)	20.84% (73,311)
Household Income as Percentage of Federal Poverty Threshold	113.74 (256.53)	135.10 (158.10)
HbA1c, %	7.64 (1.94)	7.86 (2.20)
Systolic Blood Pressure, mm Hg	130.01 (13.52)	132.22 (19.48)
Diastolic Blood Pressure, mm Hg	78.23 (7.97)	78.30 (11.10)
LDL Cholesterol, mg/dL	101.07 (35.24)	100.17 (38.12)

For individuals who underwent housing instability assessment, values for time varying variables are relative to the date of first housing stability assessment. For individuals who did not undergo housing stability assessment, values for time varying variables are from a randomly selected date

HbA1c = hemoglobin a1c; LDL = low density lipoprotein

'Not Reported' for the racial identity variables indicates that a racial identity was not reported in the EHR data used for this study

© 2025 Berkowitz SA et al. *JAMA Network Open*.

eTable 3. Estimated Mean Outcomes at Study Time Points Under Different Counterfactual Scenarios

Outcome	Time Point, months	Counterfactual Scenario	Estimate	Lower 95% Confidence Limit	Upper 95% Confidence Limit
Hemoglobin A1c	6	No Housing Instability	7.58	7.56	7.59
Hemoglobin A1c	6	Housing Instability	7.69	7.65	7.74
Systolic Blood Pressure	6	No Housing Instability	129.02	128.90	129.14
Systolic Blood Pressure	6	Housing Instability	129.36	129.07	129.65
Diastolic Blood Pressure	6	No Housing Instability	77.29	77.22	77.36
Diastolic Blood Pressure	6	Housing Instability	77.53	77.36	77.70
LDL Cholesterol	6	No Housing Instability	98.28	97.77	98.79
LDL Cholesterol	6	Housing Instability	98.74	97.46	100.03
Hemoglobin A1c	12	No Housing Instability	7.58	7.56	7.60
Hemoglobin A1c	12	Housing Instability	7.70	7.66	7.75
Systolic Blood Pressure	12	No Housing Instability	129.24	129.10	129.38
Systolic Blood Pressure	12	Housing Instability	130.01	129.65	130.36
Diastolic Blood Pressure	12	No Housing Instability	77.29	77.21	77.37
Diastolic Blood Pressure	12	Housing Instability	77.56	77.36	77.77
LDL Cholesterol	12	No Housing Instability	96.71	96.18	97.25
LDL Cholesterol	12	Housing Instability	98.18	96.78	99.57
Hemoglobin A1c	18	No Housing Instability	7.59	7.57	7.61
Hemoglobin A1c	18	Housing Instability	7.72	7.67	7.78
Systolic Blood Pressure	18	No Housing Instability	128.90	128.74	129.06
Systolic Blood Pressure	18	Housing Instability	129.81	129.38	130.24
Diastolic Blood Pressure	18	No Housing Instability	77.01	76.91	77.10

© 2025 Berkowitz SA et al. *JAMA Network Open*.

Diastolic Blood Pressure	18	Housing Instability	77.52	77.27	77.77
LDL Cholesterol	18	No Housing Instability	97.16	96.59	97.73
LDL Cholesterol	18	Housing Instability	97.02	95.43	98.60
Hemoglobin A1c	24	No Housing Instability	7.61	7.59	7.63
Hemoglobin A1c	24	Housing Instability	7.73	7.68	7.79
Systolic Blood Pressure	24	No Housing Instability	128.63	128.46	128.80
Systolic Blood Pressure	24	Housing Instability	130.08	129.63	130.54
Diastolic Blood Pressure	24	No Housing Instability	76.83	76.73	76.93
Diastolic Blood Pressure	24	Housing Instability	77.32	77.06	77.59
LDL Cholesterol	24	No Housing Instability	96.87	96.25	97.49
LDL Cholesterol	24	Housing Instability	98.84	97.15	100.53
Hemoglobin A1c	30	No Housing Instability	7.58	7.55	7.60
Hemoglobin A1c	30	Housing Instability	7.65	7.58	7.71
Systolic Blood Pressure	30	No Housing Instability	128.19	127.96	128.41
Systolic Blood Pressure	30	Housing Instability	127.76	127.11	128.40
Diastolic Blood Pressure	30	No Housing Instability	76.38	76.24	76.52
Diastolic Blood Pressure	30	Housing Instability	76.60	76.23	76.97
LDL Cholesterol	30	No Housing Instability	95.38	94.59	96.17
LDL Cholesterol	30	Housing Instability	95.50	94.08	96.92
Hemoglobin A1c	36	No Housing Instability	7.59	7.56	7.61
Hemoglobin A1c	36	Housing Instability	7.78	7.64	7.92
Systolic Blood Pressure	36	No Housing Instability	128.25	128.02	128.49
Systolic Blood Pressure	36	Housing Instability	129.28	128.01	130.54

© 2025 Berkowitz SA et al. *JAMA Network Open*.

Diastolic Blood Pressure	36	No Housing Instability	75.96	75.82	76.10
Diastolic Blood Pressure	36	Housing Instability	76.64	75.95	77.32
LDL Cholesterol	36	No Housing Instability	95.70	94.81	96.59
LDL Cholesterol	36	Housing Instability	97.81	93.86	101.76

Mean outcomes under different counterfactual scenarios were estimated using a longitudinal targeted minimum loss estimation approach.
LDL = Low Density Lipoprotein
Hemoglobin A1c is reported in percentage points, systolic and diastolic blood pressure is reported in mm Hg, and LDL cholesterol is reported in mg/dL

© 2025 Berkowitz SA et al. *JAMA Network Open*.

eTable 4. Comparison of Results at 12 Months Estimated Using SuperLearner and Generalized Linear Models

Estimate	SuperLearner Result*	Generalized Linear Model Result*
Hemoglobin A1c		
Mean Hemoglobin A1c If No Housing Instability, %	7.57 (7.56 to 7.59)	7.58 (7.56 to 7.60)
Mean Hemoglobin A1c If Housing Instability, %	7.69 (7.64 to 7.75)	7.70 (7.66 to 7.75)
Estimated Difference in Means for Hemoglobin A1c, %	-0.12 (-0.18 to -0.06)	-0.12 (-0.17 to -0.07)
Systolic Blood Pressure		
Mean Systolic Blood Pressure If No Housing Instability, mm Hg	7.57 (7.56 to 7.59)	129.24 (129.10 to 129.38)
Mean Systolic Blood Pressure If Housing Instability, mm Hg	7.69 (7.64 to 7.75)	130.01 (129.66 to 130.37)
Estimated Difference in Means for Systolic Blood Pressure, mm Hg	-0.12 (-0.18 to -0.06)	-0.77 (-1.14 to -0.39)
Diastolic Blood Pressure		
Mean Diastolic Blood Pressure If No Housing Instability, mm Hg	77.30 (77.22 to 77.38)	77.29 (77.21 to 77.37)
Mean Diastolic Blood Pressure If Housing Instability, mm Hg	77.49 (77.25 to 77.73)	77.56 (77.35 to 77.76)
Estimated Difference in Means for Diastolic Blood Pressure, mm Hg	-0.19 (-0.43 to 0.06)	-0.27 (-0.48 to -0.05)
LDL Cholesterol		
Mean LDL Cholesterol If No Housing Instability, mg/dL	96.81 (96.30 to 97.31)	96.74 (96.21 to 97.28)
Mean LDL Cholesterol If Housing Instability, mg/dL	98.10 (96.44 to 99.76)	98.11 (96.73 to 99.48)
Estimated Difference in Means for LDL Cholesterol, mg/dL	-1.29 (-2.99 to 0.41)	-1.36 (-2.83 to 0.10)

Estimated difference in mean compares the estimated difference in the outcomes between counterfactual scenarios in which individuals did not versus did experience housing instability from the time of first assessment to the 12-month time point. A negative value indicates estimated benefit for preventing housing instability. The differences in means were estimated using a longitudinal targeted minimum loss estimation approach. Approaches used for estimation via SuperLearner were: generalized linear models, multivariate adaptive regression splines, and gradient boosted trees.
*Owing to computational intensity of the SuperLearner analyses, only 10 multiple imputation datasets were used for these analyses, in contrast to 50 datasets used in the main analyses (e.g., as presented in Table 2). We used the same 10 imputation datasets for both the SuperLearner and generalized linear model analyses presented in this table for comparability.
LDL = Low Density Lipoprotein.

© 2025 Berkowitz SA et al. *JAMA Network Open*.

eTable 5. Demographic Characteristics of Cohort that Reported Housing Instability and Were Reassessed within 12 Months

Characteristic	Overall	Did Not Report Housing Instability	Reported Housing Instability	P-value
	N = 3718	N = 2154	N = 1564	
	Mean (SD) or % (N)	Mean (SD) or % (N)	Mean (SD) or % (N)	
Age at Initial Housing Instability Assessment, years	53.97 (11.94)	54.64 (12.35)	53.06 (11.31)	<0.001
Female	53.78% (1,999)	56.64% (1,220)	49.84% (779)	<0.001
Racial Identity				<0.001
American Indian/Alaska Native	1.86% (69)	2.04% (44)	1.60% (25)	
Asian	3.12% (116)	4.27% (92)	1.53% (24)	
Black	34.78% (1,293)	34.45% (742)	35.23% (551)	
Multiple	1.37% (51)	1.11% (24)	1.73% (27)	
Native Hawaiian or Other Pacific Islander	0.94% (35)	1.02% (22)	0.83% (13)	
Not Reported	6.80% (253)	6.50% (140)	7.23% (113)	
White	51.13% (1,901)	50.60% (1,090)	51.85% (811)	
Hispanic Ethnicity	33.98% (1,223)	37.19% (777)	29.54% (446)	<0.001
Primary Language Other Than English	32.36% (1,202)	38.12% (821)	24.41% (381)	<0.001
Comorbidity Index	0.28 (0.91)	0.25 (0.93)	0.34 (0.89)	<0.001
Health Insurance				<0.001
Medicaid	47.66% (1,762)	45.92% (986)	50.06% (776)	
Medicare	21.61% (799)	23.89% (513)	18.45% (286)	
Other Public	2.87% (106)	3.17% (68)	2.45% (38)	
Private	9.76% (361)	11.46% (246)	7.42% (115)	
Uninsured	18.10% (669)	15.56% (334)	21.61% (335)	
Household Income as Percentage of Federal Poverty Threshold	70.49 (224.90)	76.87 (264.34)	62.03 (157.64)	<0.001
Social Vulnerability Index at Census Tract Level	0.71 (0.25)	0.72 (0.25)	0.70 (0.26)	0.091
Mean HbA1c in Year Prior to Housing Instability Assessment, %	7.79 (2.12)	7.64 (1.97)	8.00 (2.31)	0.002
Mean Systolic Blood Pressure in Year Prior to Housing Instability Assessment, mm Hg	129.20 (13.17)	128.95 (12.66)	129.56 (13.85)	0.59
Mean Diastolic Blood Pressure in Year Prior to Housing Instability Assessment, mm Hg	78.99 (7.99)	78.54 (7.79)	79.63 (8.24)	<0.001
Mean LDL Cholesterol in Year Prior to Housing Instability Assessment, mg/dL	102.38 (34.94)	102.07 (34.52)	102.87 (35.60)	0.62

© 2025 Berkowitz SA et al. *JAMA Network Open*.

Greater comorbidity index scores indicate greater comorbidity; greater social vulnerability index scores indicate greater risk; HbA1c = hemoglobin a1c; LDL = low density lipoprotein

© 2025 Berkowitz SA et al. *JAMA Network Open*.

eTable 6. Characteristics of Cohort Reporting Housing Instability and Reassessed within 12 Months Compared with Those Not Reassessed within 12 Months

Characteristic	Re-assessed Within 12 Months	Not Re-assessed Within 12 Months
	N = 3718	N = 14602
	Mean (SD) or % (N)	Mean (SD) or % (N)
Age at Initial Housing Instability Assessment, years	53.97 (11.94)	52.80 (11.88)
Female	53.78% (1,999)	52.34% (7,642)
Racial Identity		
American Indian/Alaska Native	1.86% (69)	1.39% (203)
Asian	3.12% (116)	2.05% (299)
Black	34.78% (1,293)	27.33% (3,991)
Multiple	1.37% (51)	1.29% (189)
Native Hawaiian or Other Pacific Islander	0.94% (35)	0.81% (118)
Not Reported	6.80% (253)	8.38% (1,223)
White	51.13% (1,901)	58.75% (8,579)
Hispanic Ethnicity	33.98% (1,223)	30.04% (4,387)
Primary Language Other Than English	32.36% (1,202)	26.94% (3,924)
Comorbidity Index	0.28 (0.91)	0.27 (0.88)
Health Insurance		
Medicaid	47.66% (1,762)	43.30% (6,182)
Medicare	21.61% (799)	18.68% (2,667)
Other Public	2.87% (106)	2.21% (3,16)
Private	9.76% (361)	10.28% (1,467)
Uninsured	18.10% (669)	25.53% (3,645)
Household Income as Percentage of Federal Poverty Threshold	70.49 (224.90)	87.00 (184.02)
Social Vulnerability Index at Census Tract Level	0.71 (0.25)	0.71 (0.23)
Mean HbA1c in Year Prior to Housing Instability Assessment, %	7.79 (2.12)	7.89 (2.15)
Mean Systolic Blood Pressure in Year Prior to Housing Instability Assessment, mm Hg	129.20 (13.17)	130.23 (14.21)
Mean Diastolic Blood Pressure in Year Prior to Housing Instability Assessment, mm Hg	78.99 (7.99)	79.25 (8.32)
Mean LDL Cholesterol in Year Prior to Housing Instability Assessment, mg/dL	102.38 (34.94)	102.13 (35.82)

Greater comorbidity index scores indicate greater comorbidity; greater social vulnerability index scores indicate greater risk; HbA1c = hemoglobin a1c; LDL = low density lipoprotein

This table allows comparison of individuals who reported housing instability but were not re-assessed and thus could not be included in the 'treatment' analyses reported in Table 3 with those who were included. This can help readers determine the extent to which those who were included may or may not have been representative of the larger cohort who reported housing instability, along with the extent to which results may be generalizable to that cohort.

© 2025 Berkowitz SA et al. *JAMA Network Open*.

eTable 7. Estimated Differences in Means under the Prevention Strategy for Individuals Prescribed Insulin

Timepoint	Hemoglobin A1c		Systolic Blood Pressure		Diastolic Blood Pressure		LDL Cholesterol	
	Estimated Difference in Mean, % (95% CI)	p-value	Estimated Difference in Mean, mm Hg (95% CI)	p-value	Estimated Difference in Mean, mm Hg (95% CI)	p-value	Estimated Difference in Mean, mg/dL (95% CI)	p-value
6 Months	-0.11 (-0.20 to -0.01)	0.03	0.10 (-0.52 to 0.72)	0.76	0.04 (-0.30 to 0.38)	0.82	0.17 (-2.62 to 2.96)	0.90
12 Months	0.03 (-0.08 to 0.14)	0.58	-0.54 (-1.36 to 0.27)	0.19	0.10 (-0.34 to 0.53)	0.67	-2.79 (-5.79 to 0.21)	0.07
18 Months	-0.22 (-0.35 to -0.10)	0.0004	-0.36 (-1.32 to 0.60)	0.46	-0.28 (-0.80 to 0.23)	0.28	0.37 (-3.24 to 3.97)	0.84
24 Months	-0.09 (-0.22 to 0.04)	0.17	-1.62 (-2.75 to -0.50)	0.005	0.07 (-0.54 to 0.68)	0.83	-0.47 (-4.15 to 3.21)	0.80
30 Months	-0.23 (-0.37 to -0.10)	0.0006	1.88 (0.60 to 3.16)	0.004	-0.14 (-0.81 to 0.53)	0.68	3.64 (0.54 to 6.75)	0.02
36 Months	-0.46 (-0.70 to -0.22)	0.0002	0.34 (-1.61 to 2.29)	0.73	-1.25 (-2.61 to 0.11)	0.07	2.94 (-2.29 to 8.16)	0.27

Estimated difference in mean compares the estimated difference in the outcomes between counterfactual scenarios in which individuals did not versus did experience housing instability from the time of first assessment to the specified time point. A negative value indicates estimated benefit for preventing housing instability. The differences in means were estimated using a longitudinal targeted minimum loss estimation approach.
LDL = Low Density Lipoprotein
CI = Confidence Interval

© 2025 Berkowitz SA et al. *JAMA Network Open*.

eTable 8. Estimated Differences in Means under the Prevention Strategy with Loss to Follow-Up Window Shortened to 12 Months

Timepoint	Hemoglobin A1c		Systolic Blood Pressure		Diastolic Blood Pressure		LDL Cholesterol	
	Estimated Difference in Mean, % (95% CI)	p-value	Estimated Difference in Mean, mm Hg (95% CI)	p-value	Estimated Difference in Mean, mm Hg (95% CI)	p-value	Estimated Difference in Mean, mg/dL (95% CI)	p-value
6 Months	-0.12 (-0.16 to -0.07)	<.0001	-0.34 (-0.64 to -0.04)	0.03	-0.24 (-0.41 to -0.06)	0.009	-0.46 (-1.84 to 0.91)	0.51
12 Months	-0.12 (-0.17 to -0.07)	<.0001	-0.77 (-1.14 to -0.39)	0.0001	-0.27 (-0.49 to -0.06)	0.01	-1.46 (-2.96 to 0.03)	0.05
18 Months	-0.11 (-0.20 to -0.01)	0.02	-1.28 (-2.03 to -0.54)	0.001	-0.70 (-1.15 to -0.24)	0.003	-0.81 (-2.73 to 1.12)	0.41
24 Months	-0.17 (-0.32 to -0.03)	0.02	-2.58 (-4.00 to -1.17)	0.0004	-0.96 (-1.70 to -0.21)	0.01	-0.24 (-4.46 to 3.97)	0.91
30 Months	-0.22 (-0.36 to -0.07)	0.004	0.59 (-0.72 to 1.90)	0.38	-0.65 (-1.50 to 0.21)	0.14	1.21 (-2.21 to 4.63)	0.49
36 Months	-0.38 (-0.68 to -0.07)	0.01	-0.82 (-1.81 to 0.16)	0.10	0.74 (-0.16 to 1.64)	0.11	-4.25 (-7.55 to -0.96)	0.01

In these sensitivity analyses, individuals who did not have housing stability assessments at least every 12 months were censored (in the main analyses, individuals who did not have assessments at least every 24 months were censored).
Estimated difference in mean compares the estimated difference in the outcomes between counterfactual scenarios in which individuals did not versus did experience housing instability from the time of first assessment to the specified time point. A negative value indicates estimated benefit for preventing housing instability. The differences in means were estimated using a longitudinal targeted minimum loss estimation approach.
LDL = Low Density Lipoprotein
CI = Confidence Interval

© 2025 Berkowitz SA et al. *JAMA Network Open*.

eTable 9. Estimated Differences in Means under the Prevention Strategy with Adjustment for Food and Transportation Needs

Timepoint	Hemoglobin A1c		Systolic Blood Pressure		Diastolic Blood Pressure		LDL Cholesterol	
	Estimated Difference in Mean, % (95% CI)	p-value	Estimated Difference in Mean, mm Hg (95% CI)	p-value	Estimated Difference in Mean, mm Hg (95% CI)	p-value	Estimated Difference in Mean, mg/dL (95% CI)	p-value
6 Months	-0.09 (-0.15 to -0.03)	0.01	-0.24 (-0.63 to 0.16)	0.24	-0.22 (-0.44 to 0.01)	0.06	-0.50 (-2.33 to 1.33)	0.59
12 Months	-0.09 (-0.16 to -0.01)	0.02	-0.54 (-1.03 to -0.06)	0.03	-0.17 (-0.46 to 0.12)	0.26	-1.23 (-3.29 to 0.83)	0.24
18 Months	-0.11 (-0.20 to -0.03)	0.01	-0.62 (-1.25 to 0.01)	0.05	-0.38 (-0.74 to -0.02)	0.04	-0.25 (-2.78 to 2.27)	0.84
24 Months	-0.10 (-0.18 to -0.01)	0.03	-1.08 (-1.81 to -0.35)	0.004	-0.38 (-0.74 to -0.02)	0.04	-4.50 (-7.49 to -1.52)	0.003
30 Months	0.05 (-0.07 to 0.17)	0.39	-0.67 (-2.08 to 0.73)	0.35	-0.77 (-1.55 to 0.01)	0.05	0.92 (-2.20 to 4.05)	0.56
36 Months	-0.02 (-0.20 to 0.16)	0.83	-1.22 (-3.63 to 1.19)	0.32	-0.36 (-1.51 to 0.78)	0.53	-0.40 (-6.36 to 5.56)	0.90

In these sensitivity analyses, we adjusted for food and transportation needs, in addition to all the factors adjusted for in the main analysis.

© 2025 Berkowitz SA et al. *JAMA Network Open*.
