# Supplementary material for: Housing Instability and Type 2 Diabetes Outcomes
Source: JAMA Netw Open. 2025 Apr 14;8(4):e254852. doi: 10.1001/jamanetworkopen.2025.4852 (PMC11997728; doi:10.1001/jamanetworkopen.2025.4852)
Supplement: Supplement 2. — Data Sharing Statement [file jamanetwopen-e254852-s002.pdf]

Data Sharing Statement

Berkowitz. Housing Instability and Type 2 Diabetes Outcomes. *JAMA Netw Open*. Published April 14, 2025. doi:10.1001/jamanetworkopen.2025.4852

Data
Data available: No

Additional Information
Explanation for why data not available: The data use agreement under which this study was conducted do not permit sharing of individual patient data
